# Aqueous and Ethanolic *Valeriana officinalis* Extracts Change the Binding of Ligands to Glutamate Receptors

**DOI:** 10.1155/2011/891819

**Published:** 2010-12-02

**Authors:** Lisa M. Del Valle-Mojica, José M. Cordero-Hernández, Giselle González-Medina, Igmeris Ramos-Vélez, Nairimer Berríos-Cartagena, Bianca A. Torres-Hernández, José G. Ortíz

**Affiliations:** Department of Pharmacology and Toxicology, School of Medicine, University of Puerto Rico, Medical Sciences Campus, P.O. Box 365067, San Juan, 00936-5067 Puerto Rico, USA

## Abstract

The effects of two valerian extracts (aqueous and hydroalcoholic) were investigated through [^3^H]Glutamate ([^3^H]Glu) and [^3^H]Fluorowillardine ([^3^H]FW) receptor binding assays using rat synaptic membranes in presence of different receptor ligands. In addition, the extract stability was monitored spectrophotometrically. Both extracts demonstrated interaction with ionotropic glutamate receptors (iGluRs). However, the extracts displayed considerable differences in receptor selectivity. The hydroalcoholic extract selectively interacted with quisqualic acid (QA), group I metabotropic glutamate receptor (mGluR) ligand, while the aqueous extract did not alter the binding of QA. The stability of the extracts was examined during several weeks. Freshly prepared extract inhibited 38–60% of [^3^H]FW binding (AMPA). After 10 days, the aqueous extract inhibited 85% of [^3^H]FW binding while the hydroalcoholic extract markedly potentiated (200%) [^3^H]FW binding to AMPA receptors. Thus, our results showed that factors such as extraction solvent and extract stability determine the selectivity for glutamate receptor (GluR) interactions.

## 1. Introduction

*Valeriana officinalis*, commonly known as Valerian, is one of approximately 250 Valerian species from the Valerianaceae family, and it is used for the preparation of phytomedicines with sedative, anxiolytic, and spasmolytic properties. The therapeutic benefits of Valerian have always been in controversy due to the inconsistent clinical results [1–3]. The discrepancies among clinical trials may be due to methodological limitations and differences in the Valerian preparations (method of extraction and extraction solvent). Many studies use water as the extraction solvent because traditionally natural product preparations (i.e., tea) are prepared in water. Aqueous extracts of Valerian have different spectrum of active constituents than extracts that have been processed with ethanol [4].

The specific mechanism(s) of action responsible for the pharmacological effects of Valerian root extracts have not been fully elucidated although it is thought that Valerian root extracts stimulate the GABA transmission [2, 3, 5–10]. On the other hand, few studies have investigated the effects of *Valeriana officinalis* in the excitatory glutamate-mediated neurotransmission [11]. 

Based on these studies, the possible effects of Valerian extracts on glutamate-mediated (excitatory) neurotransmission were assessed using receptor binding assays with rat synaptic membranes in presence of aqueous and hydroalcoholic Valerian extracts and glutamate receptors ligands (MK-801 for NMDA, AMPA or [^3^H]Fluorowillardiine for AMPA, kainic acid for KA, and quisqualic acid (QA) for Group I metabotropic glutamate receptors (mGluRs)).

## 2. Materials and Methods

### 2.1. Chemicals

 L-[2,3,4-^3^H]-Glutamic acid (60 Ci/mmol) and [^3^H]Fluorowiillardiine (80 Ci/mmol) were obtained from American Radiolabeled Chemicals, Inc (St. Louis, MO). AMPA (*α*-amino-3-hydroxyl-5-methyl-4-isoxazole-propionic acid) was obtained from Tocris Cookson, Inc. (Ellisville, MO). Potassium Chloride was purchased from Matheson Coleman & Bell (Norwood, OH). UniverSol ES was obtained from MP Biomedicals (Solon, OH). All other reagents were obtained from Sigma Chemical Company (St. Louis, MO).

### 2.2. Valerian Extracts

 Organically grown and certified Valerian dry powdered roots (Lot. 1111H-OUP), harvested in 2004, were obtained from Pacific Botanicals (LLC Grants Pass, Oregon). *Commercial Valerian*. Nature's Resources Extract (Lot. LD11282N) and Nature's Resource Herb (Lot. MG11258) were purchased from a local pharmacy. Valerian was extracted in ultra pure water or ethyl alcohol (EtOH) 70% (1 : 10 w/v) at ~23°C, stirred for 1 hour, and filtered through a 12.5 cm Whatman Qualitative no. 1 filter. Aliquots were centrifuged at 6,700 g to remove particulates and stored at 4°C.

### 2.3. Cerebral Cortex Synaptic Membranes

Analytical Biological Services, Inc. (Wilmington, DE) prepared the synaptic membranes as follows: female rats of approximately two months of age were decapitated and the brain promptly removed. The cortex was dissected and homogenized (1 : 10 w/v) in ice-cold 10 mM Tris-HCl buffer pH 7.4. The homogenate was centrifuged twice at 2,500 g for 10 min. The resulting supernatant was centrifuged at 12,500 g for 20 min. The pellet was washed twice with ice-cold 10mM Tris-HCl buffer pH 7.4 (1 : 10 w/v) and centrifuged at 12,500 g for 20 min. The pellet (synaptical membrane, P2) was resuspended in 10 mM Tris-HCl buffer pH 7.4 and freeze thawed at least three times before being stored at −80°C until used. Protein concentration was determined using the Bradford assay [12] using bovine serum albumin (BSA) as reference standard. 

### 2.4. Receptor Binding Assays

#### 2.4.1. [^3^H]Glutamate Binding

Receptor binding competition assays were done using synaptic membranes of cerebral cortex from Analytical Biological Services, Inc. (Wilmington, DE). The reaction was initiated by the addition of tissue (100 *μ*g protein) to tubes containing 1 mM of (MK-801 for NMDA, AMPA, kainic acid for KA, and quisqualic acid (QA) for Group I metabotropic glutamate receptors (mGluR) and 20 nM [^3^H]Glutamic Acid ([^3^H]Glu) in a final volume of 500 *μ*L of 50 mM Tris HCl/100 mM KCl buffer, pH 7.4). The nonspecific binding was determined in the presence of 1 mM nonradioactive glutamate. Total binding was determined in the presence of Valerian extracts in different solvents (H_2_O and EtOH) in order to do competition studies. All samples were incubated on ice (0–4°C) for 40 minutes. The assay was stopped by centrifugation for 30 min at 6,764 g; the supernatant was extracted and the pellet washed two times with 1 mL of ice-cold buffer. The pellet was resuspended in 500 *μ*L of buffer. Radioactivity of the samples was quantified in a Beckman LS 6500 Multipurpose Scintillation Counter with 1 mL of UniverSol ES scintillation cocktail. Results are shown as percentage of total binding (±SEM).

#### 2.4.2. [^3^H]Fluorowillardine Binding

 Assays were done using synaptic membranes of cerebral cortex from Analytical Biological Services, Inc. (Wilmington, DE). The reaction was initiated by the addition of tissue (100 *μ*g protein) to tubes containing 15 nM [^3^H] Fluorowillardine ([^3^H]FW) in a final volume of 500 *μ*L of 50 mM Tris HCl/100 mM KCl buffer, pH 7.4. Nonspecific binding was determined in the presence of 10 mM glutamate. Total binding was determined in the presence of Valerian extracts in different solvents (H_2_O and EtOH) in order to do competition studies. All samples were incubated on ice (0–4°C) for 40 minutes. The assay was stopped by centrifugation for 30 min at 6,764 g. Then, the supernatant was removed and the pellet washed two times with 1 mL of ice-cold buffer. The pellet was resuspended in 500 *μ*L of buffer. Radioactivity of the samples was quantified in a Beckman LS 1800 counter with 1 mL of UniverSol scintillation cocktail. Results are shown as percentage of total binding (±SEM).

### 2.5. Wavelength Scans

 Spectrophotometric analyses were done with a Beckman DU Series 500 Spectrophotometer (Fullerton, CA). A volume of 1 mL of 10 mg/mL Valerian extract was placed in Elkay Ultra-Vu Disposable Cuvettes from Elkay Products, Inc. (Shrewsbery, MA). The scans were done from 190 nm to 320 nm in steps of 1.0 nm.

### 2.6. Statistical Analysis

Data are expressed as mean values ± the standard error of the mean (SEM) of at least three experiments. The differences between the experimental groups were tested for significance using one way analysis of variance followed by Tukey-Kramer Multiple Comparisons Test, with *P* < .05. Statistics for the experimental group versus total binding are not shown for clarity. 

## 3. Results

### 3.1. Extraction Solvents Change Ligand Binding to Glutamate Receptors

**Figure 1:**
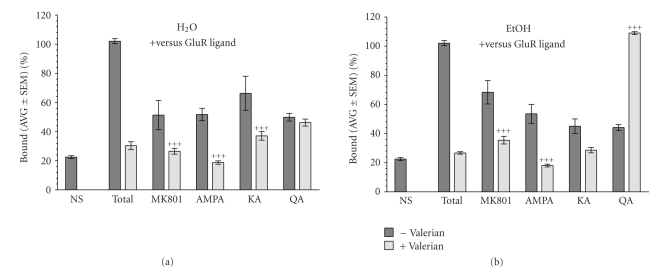
Effects of different fresh Valerian extracts (1 mg/mL) on [^3^H]Glutamate binding to rat synaptic membranes in the presence of 1 mM glutamate receptor (GluR) ligands. (a) The aqueous extract decreases the effects of the NMDA (MK-801), AMPA, and Kainate (KA), but it did not alter the effects of quisqualic acid (QA), a group I metabotropic glutamate receptor (mGluR) ligand. (b) The hydroalcoholic extract also decreases the effects of NMDA, AMPA, and KA. In contrast, Valerian reverses the inhibitory effects of QA. ^+^ *agonist versus agonist *+* Valerian, P* < .05; ^++^ *P* < .01; ^+++^ *P* < .001.

Figure 1 shows the effects of different Valerian extracts (1 mg/mL) on [^3^H]Glutamate binding to rat synaptic membranes in the presence of 1 mM GluR ligands (NMDA, AMPA, KA, or QA). The aqueous extract (a) decreases the effects of the NMDA (MK-801), AMPA, and kainate (KA), but it did not alter the effects of quisqualic acid (QA), a Group I metabotropic glutamate receptor (mGluR) ligand. The hydroalcoholic extract (b) has similar effects as the aqueous extracts on NMDA, AMPA, and KA treated membranes. However, the hydroalcoholic extract potentiates the effects of QA, suggesting a strong effect on group I mGluR.

**Figure 2:**
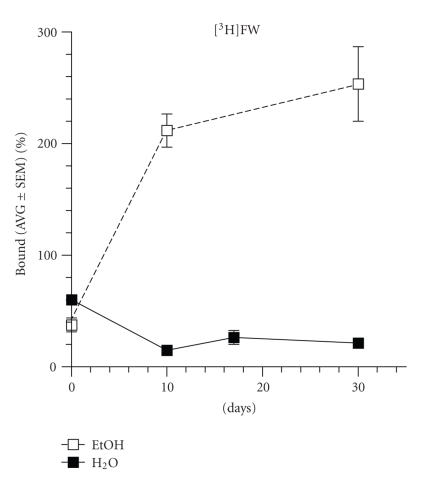
Changes in ^3^[H]Fluorowillardine binding with time. Initially, a 60% of inhibition is observed with the aqueous extracts while the hydroalcoholic extract inhibits only a 38%. However, with time, the inhibitory effects of the aqueous extract increase to 85% whereas the hydroalcoholic extract markedly potentiates (200%) ^3^[H]Fluorowillardine ([^3^H]FW) binding to AMPA receptors.

Similarly, the effects of aqueous and hydroalcoholic extracts on AMPA were evaluated by binding assays using [^3^H]FW. Initially, the aqueous and hydroalcoholic extracts (Figure 2) have 60% and 38% inhibitory effects on [^3^H]FW binding, respectively. However, with time (4 weeks), the aqueous extract inhibits [^3^H]FW to 85% while the hydroalcoholic extract markedly increases [^3^H]FW binding (200%) to AMPA receptors.

### 3.2. Extract Stability Alters Ligand Binding to Glutamate Receptor

**Figure 3:**
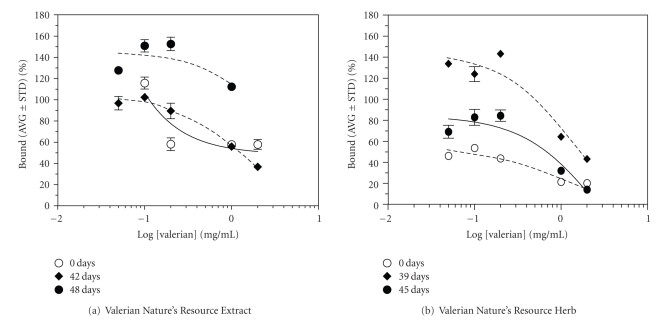
Effect of time in ^3^[H]Fluorowillardine binding from two Valerian commercial products. (a) At time zero, aqueous extracts of Nature's Resource Extract and Nature's Resource Herb inhibit [^3^[H]]Fluorowillardine binding. (b) After a period of time (42 days (Extract) or 39 days (Herb)), the extracts loose the inhibitory effects. Further in time (48 days (Extract) or 45 days (Herb)), they potentiate [^3^[H]]Fluorowillardine binding.

The effect of two Valerian commercial products (prepared as aqueous extracts) was evaluated on AMPA binding ([^3^H]FW). Figure 3 shows the effect of time in [^3^H]FW binding. (a) At time zero, Valerian Nature's Resource Extract and Nature's Resource Herb inhibit [^3^H]Fluorowillardine binding. (b) After a period of time (42 days (Extract) or 39 days (Herb)), the extracts loose the inhibitory effects. Further in time (48 days (Extract) or 45 days (Herb)), they potentiate [^3^H]Fluorowillardine binding.

### 3.3. Spectrophotometric Analysis of Aqueous and Hydroalcoholic Extracts

**Figure 4:**
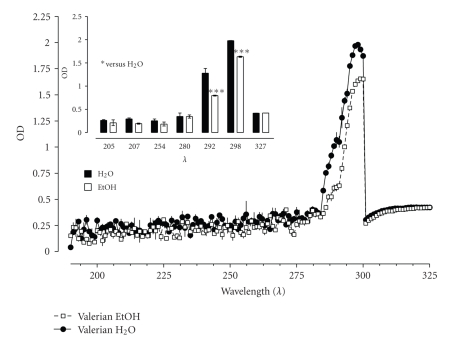
Spectra and absorbance from aqueous and hydroalcoholic Valerian extracts (10 mg/mL). Significant differences at 292 and 298 nm were observed. H_2_O* versus *EtOH, *P* < .05; ^++^ *P* < .01; ^+++^ *P* < .001.

The spectra of different Valerian extracts (10 mg/mL) prepared in H_2_O and EtOH were evaluated spectrophotometrically as shown in Figure 4. The insert in Figure 4 shows a list of wavelength and only two, 292 and 298 nm, demonstrate significant differences. 

## 4. Discussion

Valerian is a well-recognized plant used in the folkloric medicine for its sedative and anxiolytic properties [2]. Numerous studies evaluate the use of Valerian root extracts to induce and improve sleep quality [13–15] while others evaluate Valerian anxiolytic effects [16–18]. In humans, Valerian extracts reduced latency to fall asleep as effectively as small doses of benzodiazepines [19]. Although Valerian's anxiolytic properties are well documented in mice and rats [20], there is no scientific evidence that could support the anxiolytic properties of the plant in humans, due to intrinsic methodological problems such as variation in Valerian doses and preparations, number of patients, and length of treatment [1]. 

It is not fully understood which constituents of Valeriana officinalis are responsible for the pharmacological actions, but many manufacturers standardize Valerian preparations according to the valerenic acid content as recommended by the German Commission E. Studies suggested that valerenic acid is the principal active component responsible for Valerian's anxiolytic properties [21, 22]. However, other Valerian constituents such as borneol, lignans, and flavonoids (hesperidin, linarin, and 6-methylapigenin) have also been found to have anxiolytic and sedative activity [23–27]. Moreover, other Valerian species with low valerenic acid concentration (e.g., *Valeriana edulis* and *Valeriana sitchensis*) have similar pharmacological profiles [19, 28–30]. Thus, there is no evidence showing that valerenic acid is exclusively responsible for the sedative and anxiolytic effects attributed to *Valeriana officinalis*.

Circosta and colleagues [31] conducted the biological and chemical characterization of two Valerian extracts (aqueous and hydroalcoholic) and found remarkable differences in the content of valepotriates. Similar results were found by Occhiuto et al. in 2008 [32]. Both studies, from the same group of researchers, revealed that the phytochemical analysis of the hydroalcoholic extract and the aqueous extract was “similar” in the valerenic acid composition (aqueous extracts being half of the hydroalcoholic extract content), but the concentration of the valepotriates was much lower in the aqueous extract than in the hydroalcoholic extract. In the biological assays, Circosta and colleagues [31] observed a lower percent of animals with arrhythmias when the hydroalcoholic and not the aqueous extracts were given at a dose of 50 mg/kg (80% versus 60%, resp.). Similarly, Occhiuto et al. [32] reported that the concentration causing 50% inhibition of the amplitude of contractions (uterorelaxant effect) was 29.5 ± 3.40 and 68.7 ± 5.20 *μ*g/mL for the hydroalcoholic and aqueous extracts, respectively. Therefore, the biological analysis by these studies demonstrated that the Valerian extracts possess coronary dilatory, hypotensive, and bronchodilatory properties as well as relaxing effects on human myometrial muscle at different concentrations; the hydroalcoholic extracts being always more potent. These differences in potency of the hydroalcoholic and aqueous extracts may be related to differences in the extraction solvent and procedure, raw material storage conditions, growing techniques, harvesting conditions, and age of the plant, among other factors. 

**Figure 5:**
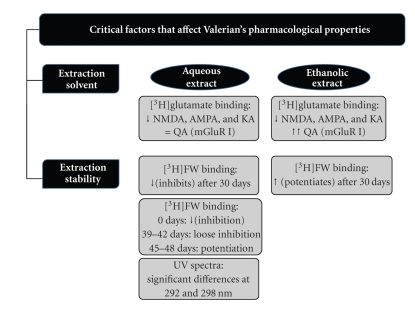
Summary of the aqueous and ethanolic Valerian extract effects in receptor binding assays and spectrophotometric absorbance.

Previously, modest effects of commercial aqueous Valerian extracts on NMDA receptor [^3^H]MK-801 binding have been reported [33]. Similarly, Malva and colleagues [11] reported AMPA-mediated neuroprotective effects of Valerian. Our experiments show that aqueous and hydroalcoholic extracts from *Valeriana officinalis* have different biological activity as demonstrated by the GluR binding assays. Aqueous and hydroalcoholic Valerian extracts interact with NMDA, AMPA, and KA receptors. Moreover, Valerian extracts effects on different receptors change with time as seen with AMPA binding assays. On the other hand, the aqueous extracts did not have effect in QA while a strong interaction with group I mGluR is observed with the hydroalcoholic extracts (see Figure 5). Previous results from our lab (not shown) had demonstrated that when valerenic acid was used, as a pure compound, in the same type of experiments, a similar response (potentiation) was obtained. These results suggested that the hydroalcoholic Valerian extract reverses the inhibitory effects of QA probably due to a higher content of valerenic acid. Further studies should be done to verify the valerenic acid content in the different extracts.

Previous studies by different researchers suggest that mGluR may have an important role in anxiety [34–36]. In particular, there is evidence involving group II mGluR (mGlu2/3) and group I mGluR (mGlu5) receptor agonists as potential anxiolytic agents [37, 38]. This mGluR role could suggest a possible mechanism by which Valerian extracts produce their effects. A more detailed study of these Valerian interactions with mGluR is being examined by our lab.

Navarrete et al. (2006) [29] used HPLC and detection at different wavelengths to identify Valerian components (i.e., chlorogenic acid, lignans, flavonoids, valerenic acids, and valepotriates) in different species. Similarly, we screened for possible spectrographic differences between the aqueous and hydroalcoholic extracts. A qualitative analysis of the absorbance spectra (at different wavelengths) of the extracts revealed significant differences at 292 and 298 nm. Further studies should be done in order to determine the relation between spectra changes and binding selectivity. 

A survey made by ConsumerLab.com reported that 4 of 17 Valerian products had no detectable Valerian compounds, four had half of the expected levels, two had lead contamination, and one had cadmium contamination (http://www.consumerlab.com) [39]. This particular situation occurred with commercial natural products manufactured by various processes using different solvents. In addition, the low stability of the compounds, mainly valepotriates, could result in the absence of these compounds in the different pharmaceutical dosage forms since they may decompose during the manufacturing process or upon storage [40].

Products containing Valerian have managed to keep their place in the natural products market, in spite of the lack of science based evidence for its efficacy and the strong competition with synthetic drugs such as benzodiazepines. Inconsistent and variable pharmacological effects of plant-derived products are one of the challenges of the natural products research. Consequently, a drug derived from natural products could be considered as a rational drug only if it is standardized and proven to meet the standards of quality (prove identity, purity, etc.). Biological screening of plant extracts instead of analysis of individual compound may be a useful tool in clarifying Valerian pharmacological effects because of possible synergism as a mechanism of action(s) of various active compounds. The present study demonstrates that Valerian extracts interact with the GluR, and factors such as the extraction solvent and stability of the extracts are critical to determine changes in selectivity for GluR interaction.

## Abbreviations

AMPA: Alpha-amino-3-hydroxy-5-methylisox-azole-4-propionic acid
BSA: Bovine serum albumin
EtOH: Ethyl alcohol
[^3^H]FW: [^3^H]Fluorowillardine
[^3^H]Glu: [^3^H]Glutamate
GluR: Glutamate receptors
Group I mGluR: Metabotropic glutamate receptor (1/5)
iGluR: Ionotropic glutamte receptor
KA: Kainic acid
mGluR: Metabotropic glutamate receptors
NMDA: N-methyl-D-aspartic acid
QA: Quisqualic acid, (2*S*)-2-amino-3-(3,5-dioxo-1,2,4-oxadiazolidin-2-yl) propanoic acid).
